# Digital Literacy and Interpersonal Trust as Predictors of Willingness to Share Patient-Generated Health Data Among Korean Internet Users: Cross-Sectional Study Using Privacy Calculus and Communication Privacy Management Theories

**DOI:** 10.2196/75448

**Published:** 2026-05-27

**Authors:** Dongsu Lee, Wonseuk Jang

**Affiliations:** 1 Future Medicine Research Center, Gangnam Severance Hospital, College of Medicine, Yonsei University, 20 63rd Road, Unju-ro, Gangnam-gu, Seoul, 06229, Republic of Korea, 82 2-2019-5442; 2 Department of Medical Device Engineering and Management, Yonsei University College of Medicine, 50-1, Yonsei-Ro, Seodaemun-guSeoul, 03722, Republic of Korea

**Keywords:** patient-generated health data, privacy, digital literacy, health data sharing, interpersonal trust, perceived risk, perceived benefit

## Abstract

**Background:**

The proliferation of wearable devices and advances in data analytics are accelerating the adoption of personalized digital health care, relying heavily on patient-generated health data (PGHD). However, the sensitive nature of this data creates significant privacy boundaries. While previous research has focused on rational cost-benefit trade-offs, there is a limited understanding of how social and cognitive factors—specifically interpersonal trust and digital literacy (DL)—shape the data-sharing decisions of the general public.

**Objective:**

This study aims to identify the factors predicting individuals’ willingness to share health data (WS) by integrating privacy calculus and communication privacy management theories. It specifically examines the comparative influence of DL, interpersonal trust, and moral motivation on data-sharing decisions.

**Methods:**

We analyzed data from the 2023 Korea Panel Survey on the Digital Society (n=4518), a nationwide representative sample of internet users. Survey-weighted structural equation modeling with weighted least squares mean and variance adjusted estimation was used to examine the relationships among perceived risk (PR), perceived benefit (PB), DL, interpersonal trust, and WS.

**Results:**

PR was the strongest negative predictor of WS (standardized coefficient [std *β*]=−0.189, *P*<.001), whereas PB was the strongest positive predictor (std *β*=0.076, *P*=.009), followed by moral motivation (std *β*=0.062, *P*=.03) and interpersonal trust. DL showed a significant negative direct association with willingness to share (std *β* = −0.060, *P*<.001). However, subdimension analysis revealed heterogeneous mechanisms: the “understanding” dimension was associated with lower PR and indirectly promoted sharing, whereas use and engagement were associated with higher PR. Age-stratified analyses suggested potential heterogeneity in these relationships, although the overall interaction was not statistically significant.

**Conclusions:**

PBs and risks were the strongest determinants of PGHD sharing, with benefits increasing and risks decreasing willingness to share. Beyond this risk-benefit balance, DL (particularly understanding) and interpersonal trust also played important roles, highlighting the need for trust-based and user-centric strategies to promote PGHD sharing and support the expansion of digital health care.

## Introduction

### Background

Due to the 4th industrial revolution, the development of big data analysis technology and the active introduction of wearable devices into the medical field are increasing the possibility of introducing personalized medicine based on individual health data and daily life data [1,2]. However, securing real-world evidence for collecting health data and measuring clinical effects is still insufficient [3]. Despite the efforts of countries to expand digital health care, research is needed regarding privacy issues, clinical usefulness has not been sufficiently proven, and the development of new services is progressing slowly due to the regulatory environment of the medical device industry [4].

To address this, research has been conducted based on various theories on the sharing of personal health data [5,6]. Previous research has mainly focused on personal health information [7,8]. However, the concept of personal health data has recently expanded beyond the scope of existing medical information to health life logs, and is developing into a concept that includes behavioral habits that affect an individual’s health [9]. Amid these changes, the concept of patient-generated health data (PGHD) is gradually expanding. Consequently, there is a growing need for research to investigate the factors influencing individuals’ willingness to share health data (WS), applying the concept of PGHD in a broader sense.

PGHD is health data that patients or the general public directly create and record using smart devices. It can play a key role in improving medical services and realizing customized health management [9]. However, the definition of PGHD is still being clarified [9,10], and the potential for using PGHD is expanding further with the spread of wearable devices. PGHD is attracting attention as an important driving force for the growth of digital health care and is recognized as an essential resource for expanding services [11,12].

PGHD, an important resource for the development of digital health care, has limitations in that it can be collected and used based on the consent of the individual who created it [13]. Recent research results have shown that the WS is known to be affected by the quality of data [12] and the purpose of use [6], and personal privacy concerns and perceived risks (PRs) have been identified as major factors that hinder the sharing of PGHD [14]. Sharing sensitive personal health data involves complex decision-making, and additional research is needed regarding privacy issues [4,5].

The willingness to share PGHD may be partially interpreted through the lens of communication privacy management (CPM) theory, which conceptualizes how individuals regulate the disclosure of private information within relational and digitally mediated contexts [15].

Private information refers to sensitive information that an individual considers to be his or her own, and includes PGHD, the subject of this study. Privacy boundaries refer to the psychological boundaries of how much an individual will share sensitive information with others, and can be a criterion for deciding whether or not to share. This study assumes that as digital literacy (DL) and interpersonal trust increase, privacy boundaries will loosen, increasing the willingness to share PGHD.

Previous studies have investigated factors that may affect the willingness to share PGHD, but most of them were limited to specific disease patient groups or small samples [4,5], and there is a lack of research that comprehensively analyzes the understanding of PGHD at the general public level and the psychological and social factors of individuals that affect the decision to share information.

In Korea, the “Korea Panel Survey on the Digital Society (KPSDS),” a national statistical survey targeting a sample of internet users, has been conducted annually since 2022 to investigate individuals’ information management behaviors, such as WS, privacy concerns, DL, and social trust, as well as the level of public awareness of informatization.

Although eHEALS (eHealth Literacy Scale) [16,17] is used as a tool to measure the ability to use health data in a specific medical environment, it is reasonable to further expand the scope of the current study by using media DL as a measurement tool to broadly survey the willingness to share PGHD to the general public, who are potential customers for future provision, as general media literacy is ultimately a basic and essential ability for using wearable devices such as smartwatches and related health services.

General internet users can be considered a representative group for studying digital health care adoption, as they are more likely to engage with technologies such as smart health care and wearable devices [18]. In order to activate PGHD sharing, which is a key resource for expanding digital health care services, it is necessary to identify factors that influence the decision to share health data when sharing sensitive health data, such as PGHD, with internet users.

### Objectives

This study aims to complement the limitations of previous studies and enhance the understanding of individuals’ willingness to share PGHD. Specifically, it analyzes the impact of key factors such as PRs and benefits, DL, and interpersonal trust on the adjustment of privacy boundaries and the formation of privacy rules, based on the CPM theory and privacy calculus theory [19].

It is differentiated from existing studies in that it uses the results of the KPSDS (2023), a national statistic for Korea, to add factors such as DL and interpersonal trust in addition to the perceived benefit (PB) and PR explained in the existing privacy calculus theory, and analyzes factors affecting the decision to share personal information by linking it with the general public’s willingness to share PGHD.

Through this, we analyze the factors influencing the decision to share personal health data for the purpose of creating a digital health care environment, additionally investigate the influence of DL and interpersonal trust on consent to share health data, seek ways to improve WS, and provide implications for establishing general policies for the expansion of digital health care services using PGHD.

### Related Work and Model Development

#### Theoretical Integration: Privacy Calculus and CPM

Privacy calculus posits that individuals make disclosure decisions by weighing PRs against PBs [20]. In health data contexts, individuals evaluate potential privacy losses—such as misuse, unauthorized access, or secondary use—against expected gains, including improved services, personalized feedback, or social value [21,22]. This framework has been widely applied in digital environments [23], social media [24], health care and mHealth settings [25-27], and e-commerce [28], consistently demonstrating that PR reduces WS, whereas PB increases it [5,29]. Accordingly, we hypothesize that PR negatively (H1) and PB positively (H2) influence willingness to share PGHD.

However, sharing PGHD involves more than rational cost-benefit evaluation. As PGHD are continuously generated, behaviorally detailed, and often highly sensitive, individuals must actively manage privacy boundaries within evolving digital ecosystems. CPM theory explains how disclosure is regulated through privacy rules shaped by relational expectations, perceived control, and trust [15,30]. CPM has been applied to health communication, wearable device use, and digital contact-tracing contexts, demonstrating how boundary coordination and trust dynamics shape disclosure decisions [19,21,24]. Rather than positioning CPM as an alternative to privacy calculus, we use it as a complementary perspective that helps interpret how individuals regulate privacy boundaries within digital health environments.

In this study, privacy calculus serves as the primary explanatory framework, while CPM is used as a complementary lens to interpret how DL and trust may shape individuals’ privacy boundary regulation.

Within the structural equation modeling framework, PR and PB represent evaluations of privacy boundary permeability, while willingness to share PGHD reflects the outcome of boundary coordination governed by privacy rules. Interpersonal trust and governance-related perceptions function as contextual factors shaping the formation and adjustment of these privacy rules.

#### Antecedents of PR and PB

Within this integrated framework, willingness to share is primarily shaped by PR and PB. Prior research suggests that several psychological and contextual factors condition these perceptions.

DL, defined as the ability to understand, evaluate, and engage with digital systems [31], may influence both risk and benefit appraisals. Higher DL can reduce uncertainty about data practices and increase perceived control, thereby lowering PR [32,33], forming the basis for H3. At the same time, DL may enhance recognition of potential advantages associated with digital health participation, increasing PB [34] and potentially strengthening WS directly [18], supporting H4 and H5.

Trust also plays a central role in shaping privacy appraisals. Interpersonal trust–generalized expectations about others’ intentions—has been shown to reduce PR in online disclosure contexts [22,24], supporting H6. It may also directly encourage sharing behavior and enhance PB [35], forming the basis for H7 and H8. Institutional trust (TR), by contrast, reflects confidence in formal governance and regulatory safeguards and is modeled primarily as an antecedent of PR.

Other contextual determinants further shape PR and PB. Privacy concern has been consistently associated with higher PR [24], whereas monetary benefit (MB) increases PB by enhancing the instrumental value of disclosure [3,36]. TR increases the perceived legitimacy of data governance, which in turn enhances the perceived societal and personal benefits of data sharing [8]. Information control (IC) and perceived effectiveness of government regulation (PE) may stabilize privacy boundaries and reduce anticipated violations, thereby lowering PR [5]. In addition, moral motivation (MM)—intrinsic intentions to contribute to socially beneficial outcomes—can increase WS by framing disclosure as prosocial and ethically meaningful [26,37].

#### Differential Effects of DL Subdimensions

DL is widely conceptualized as a multidimensional construct encompassing distinct domains of digital competence. Frameworks such as DigComp 3.0 distinguish differentiated competence areas, while empirical studies identify multiple behavioral domains of digital engagement [38,39]. Drawing on these perspectives, we conceptually organize DL into 3 competencies: digital literacy of use (DU), digital literacy of understanding (DD), and digital literacy of engagement (DE).

DU, reflecting operational skills, is associated with greater perceived control and digital self-efficacy [40]. DD, capturing critical evaluation and awareness of data practices, is linked to heightened privacy risk awareness [41]. DE, referring to active participation in digital ecosystems, is associated with social capital and PBs of interaction [42]. As these dimensions correspond to distinct psychological mechanisms—control, risk awareness, and benefit orientation—they may exert heterogeneous effects on WS. Accordingly, we examine whether DU, DD, and DE differentially influence willingness to share (H9).

#### Summary of Hypothesis Development

Integrating privacy calculus and CPM theory, the proposed model explains willingness to share PGHD primarily through PR and PB, while DL, interpersonal trust, MM, and governance-related perceptions shape these evaluations. This framework provides the theoretical basis for the following hypotheses:

- H1: PR negatively affects WS.
- H2: PB positively affects WS.
- H3: DL negatively affects PR.
- H4: DL positively affects WS.
- H5: DL positively affects PB.
- H6: interpersonal trust negatively affects PR.
- H7: interpersonal trust positively affects WS.
- H8: interpersonal trust positively affects PB.
- H9: DL subdimensions (DU, DD, and DE) exert differential effects on WS.

## Methods

### About KPSDS

#### Overview

This study is a secondary analysis of the 2023 User Panel Survey on the Intelligent Information Society, a nationwide representative longitudinal survey conducted by the Korea Information Society Development Institute (KISDI; 164004). Data were collected from October to December 2023 via 1:1 in-person computer-assisted personal interviewing interviews with 4581 daily internet users aged 15‐69 years representing an estimated population of 39,027,595.

To minimize common method variance (CMV), the original survey used procedural remedies, including respondent anonymity and the use of varied Likert-type scales (4-, 5-, and 7-point) across 7 distinct thematic sections.

#### Study Sample and Selection Bias Check

This study used data from the 2023 User Panel Survey on the Intelligent Information Society, a nationwide longitudinal survey conducted by the KISDI (164004).

The survey targeted internet users aged 15 to 69 years, representing an estimated population of 39,027,595. Responses of “no experience” (coded as 9) on PR items (q15_) were treated as missing. Participants who selected “no experience” for all PR items (63/4581, 1.38%) were excluded from the analysis because PR could not be meaningfully constructed for these cases, leaving a final analytic sample of 4518 respondents (weighted n=38,420,934).

To assess the potential impact of excluding respondents with missing PR values, we conducted sensitivity analyses using alternative imputation strategies. Missing PR values were imputed using minimum (1), median (2.5), and maximum (4) scores, and the structural model was re-estimated under each scenario. Additionally, to evaluate potential selection bias, we compared DL between excluded and included respondents using survey-weighted *t* tests.

#### Data Validity

To assess potential CMV, the Harman single-factor test was conducted at the item level using polychoric correlations to account for the ordinal nature of the data. Due to structurally missing responses (“no experience”), this supplementary test was designed to be evaluated separately for the core measurement items and the perceived-risk items.

#### Variables and Measures

In this study, variables used in previous studies were selected by using medical and health-related items from the 2023 KPSDS [5]. Detailed variable definitions, questionnaire items, and operationalization procedures are provided in Multimedia Appendix 1. For descriptive purposes, composite mean scores were reported for all variables to facilitate interpretation on the original Likert scale. However, in the structural equation modeling, PB and DL were modeled as latent constructs, whereas the remaining variables were operationalized as composite scores.

#### Dependent Variable

Regarding WS, the degree of consent and permission to share personal health data was measured using a 5-point Likert scale (q16_9, 1=absolutely not, 5=completely possible). In the structural equation modeling, WS was treated as an ordered categorical variable with 5 categories and specified as an ordered outcome in *lavaan*.

#### Independent Variables

Referring to the research design of previous studies, questions related to PR, PB, PE, MB, MM, IC, privacy concern, and TR were selected as measurement tools using the concept of MM defined in previous studies [5]. PR was measured on a 4-point Likert scale (1=always, 4=never). Responses coded as “no experience” (9) were treated as missing, and respondents who selected “no experience” for all PR items were excluded from the analysis (n=63). Valid PR items were reverse-coded so that higher scores indicate a higher frequency of PR (ie, 4=always, 1=never). All other items (PB, PE, MB, MM, IC, privacy concern, and TR) were assessed on a 5-point Likert scale (1=never, 5=completely possible) and operationalized as composite scores, except for PB, which was modeled as a latent construct.

DL was assessed using a media literacy framework rather than the conventional eHEALS, given that the creation and sharing of PGHD typically occur in a general digital environment. Therefore, general digital media usage skills were considered more relevant. A total of 23 items related to DL were rated on a 5-point Likert scale (1=“not at all” to 5=“very much”). The items were grouped into 3 subdimensions: DU (6 items, q33_1–q33_6), DD (11 items, q33_8–q33_15 and q33_19–q33_21), and DE (6 items, q33_16–q33_18 and q33_22–q33_24).

Each subdimension (DU, DD, and DE) was modeled as a first-order latent construct measured by its respective items. These first-order constructs were specified as indicators of a second-order latent construct representing overall DL. All subscales demonstrated high internal consistency, with Cronbach *α* values of 0.89 for DU, 0.93 for DD, and 0.91 for DE.

As a supplementary scale check, we also conducted an item-level CFA for the DL subdimensions (DU, DD, and DE); detailed results (including composite reliability [CR] and average variance extracted [AVE] for each subscale) are provided in Multimedia Appendix 2.

Interpersonal trust was assessed with a 7-point scale question, “How much do you generally trust people?” To address sparse distributions at extreme ends of the scale, the scale was recoded into 5 categories by combining responses 1 and 2, and 6 and 7.

#### Control Variables

Sociodemographic variables included sex, age group (AG), residential area (RA), education level, occupation, and household income (HI).

AGs were categorized into 5 groups: twenties and younger, thirties, forties, fifties, and sixty and older.

RA was classified into 3 categories: province, metropolitan city, and special city (Seoul).

Education level was recategorized into 3 groups: high school or below, college, and university or higher.

HI was classified into 4 categories: less than 2 million KRW, 2 to 4 million KRW, 4 to 6 million KRW, and 6 million KRW or more.

Additionally, perceived standard of living (SL) was included as a control variable. This was assessed by asking participants, "If we divide the living standards of our people into five levels: upper, upper-middle, middle, lower-middle, and lower, where do you think your family’s living standards fall?” on a 5-point Likert scale (1=upper, 5=low). Responses were categorized into “low” (4‐5 points) and “high” (1‐3 points) for analysis.

#### CHERRIES Checklist Mapping (Adapted)

This study is a secondary analysis of KISDI’s national panel survey; therefore, CHERRIES (Checklist for Reporting Results of Internet E-Surveys) items related to online survey administration (eg, view rate, cookies, and IP checks) are not applicable. We report available items, including survey mode, sampling, consent procedures, and data handling per the KISDI documentation.

#### Study Design

**Figure 1.**
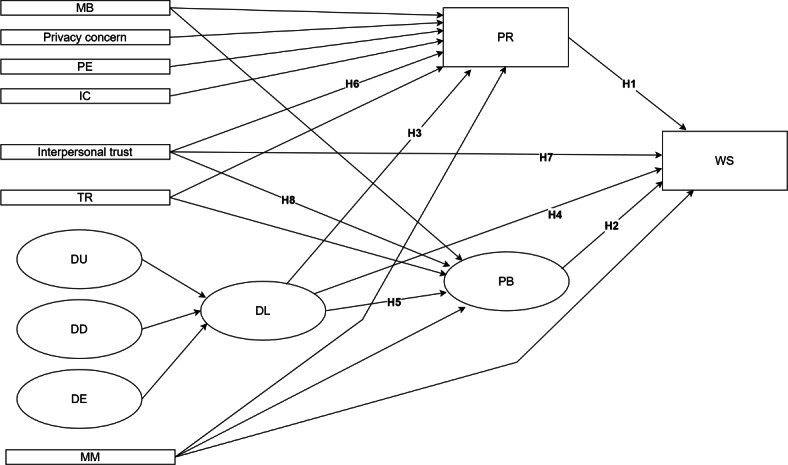
Hypothesized structural model of WS based on a cross-sectional, nationwide representative survey of Korean internet users aged 15-69 years (South Korea, 2023). DL is modeled as a latent construct indicated by DU, DD, and DE. The model includes PR, PB, interpersonal trust, TR, and MM as predictors of WS. Demographic variables (SX, RA, EL, OC, HI, SL) were analyzed descriptively and via chi-square tests (Table S15, Multimedia Appendix 2) but were not included as covariates in the SEM. DD: digital literacy of understanding; DE: digital literacy of engagement; DL: digital literacy; DU: digital literacy of use; EL: education level; HI: household income; IC: information control; MB: monetary benefit; MM: moral motivation; OC: occupation; PB: perceived benefit; PE: perceived effectiveness of government regulation; PR: perceived risk; SL: perceived standard of living; SX: sex; RA: residential area; TR: institutional trust; WS: willingness to share health data.

We conducted a cross-sectional analysis of a nationwide representative survey. The proposed research model illustrating hypothesized relationships is shown in Figure 1.

### Analysis

#### Statistical Analysis and Structural Equation Modeling Specification

All analyses were conducted in R (version 4.5.2; R Foundation) using the *lavaan* package (version 0.6.21). Descriptive statistics and chi-square tests were used to summarize sample characteristics.

We used covariance-based structural equation modeling in *lavaan* and incorporated population weights provided by the national survey through the sampling.weights argument to obtain weighted parameter estimates. As the outcome (WS) was specified as an ordered categorical variable (5 categories), models were estimated using the WLSMV (weighted least squares mean and variance adjusted; robust DWLS) estimator. Under this framework, robust (scaled) fit indices and SEs were reported, and thresholds (t1-t4) were estimated for WS.

Only PB and DL-related constructs (DL, DU, DD, and DE) were specified as latent variables. PR and all other exogenous predictors were treated as observed composite variables, and WS was modeled as an ordered observed outcome.

To assess potential multicollinearity among trust-related constructs (interpersonal trust, TR, PE, and IC), we examined survey-weighted correlations and variance inflation factors.

Both unstandardized estimates (B) and fully standardized coefficients (std *β;* std all) were reported.

#### Measurement Model Evaluation

Measurement model evaluation focused on the latent measurement structure, including PB and the hierarchical DL construct (DU, DD, and DE, forming the higher-order construct DL). PR and other composite variables were not included in this evaluation because they were operationalized as observed indices rather than reflective latent constructs. Furthermore, because model 2 retained the same first-order measurement structure as model 1, the measurement evaluation was not repeated for model 2.

The measurement model was evaluated using fully standardized factor loadings (std all). Convergent validity was assessed through CR and AVE, with recommended thresholds of CR ≥.70 and AVE ≥.50.

Discriminant validity was examined using the Fornell-Larcker criterion, whereby the square root of AVE for each construct was compared with interconstruct correlations, and the heterotrait-monotrait (HTMT) ratio, with values below 0.85 indicating adequate discriminant validity. HTMT ratios were computed based on unweighted polychoric correlations as a supplementary discriminant validity check, while the primary measurement evaluation was conducted using the survey-weighted estimation consistent with the structural model. CR, AVE, and Fornell-Larcker comparisons are reported in the Multimedia Appendix 2.

#### Model Fit Assessment

Model fit was evaluated using the robust (scaled) chi-square statistic and degrees of freedom, along with the comparative fit index (CFI), Tucker-Lewis index (TLI), root mean square error of approximation (RMSEA; 90% CI), and standardized root mean square residual (SRMR), as recommended for WLSMV estimation with ordered categorical outcomes.

Fit indices were interpreted based on established structural equation modeling guidelines [43,44]. As the chi-square statistic is sensitive to large sample sizes, greater emphasis was placed on incremental and residual-based fit indices (CFI, TLI, RMSEA, and SRMR) rather than on chi-square alone.

### Structural Models

#### Overview

Sociodemographic variables, including RA, HI, and perceived SL, were not included as covariates in the primary structural equation modeling. The proposed model was specified as a theory-driven framework focusing on proximal psychological and perceptual mechanisms, such as PR and PB. Consistent with this perspective, sociodemographic factors were conceptualized as distal variables whose effects on data-sharing intentions may be mediated through these core psychological constructs.

We specified 2 structural equation models to address the hypothesized pathways (model 1) and differential subcomponent effects (model 2).

#### Model 1 (Hypothesized Structural Model)

This was specified as a theory-driven baseline structural model to test the hypothesized pathways (H1-H8).

DL was modeled as a second-order latent construct reflected by three first-order latent constructs: DU, DD, and DE. Each first-order construct was measured using its corresponding survey items (q33 series).

Interpersonal trust and DL were specified as predictors of PR and PB, which in turn predicted WS. MM was included as an exogenous theoretical predictor with direct effects on PR, PB, and WS.

Additional observed exogenous predictors—including MB, privacy concern, PE, IC, TR, and MM—were incorporated into the structural equations as specified in Figure 1.

All model specifications were theory-driven and transparently reported in the final path diagram and supplementary material.

#### Model 2 (H9: Differential Effects of DL Subcomponents)

To test hypothesis 9, the higher-order DL construct was removed, and its 3 first-order latent subdimensions (DU, DD, and DE) were retained and modeled separately as predictors of PR, PB, and WS, allowing direct comparison of their differential effects. To account for the conceptual relatedness among the DL subdimensions, 3 residual covariances (DU~~DD, DD~~DE, and DE~~DU) were specified a priori. This specification was theoretically justified because these subdimensions represent related and interdependent facets of the broader multidimensional construct of DL, and shared variance among them may remain unaccounted for when the higher-order factor is not explicitly modeled.

We estimated (1) direct effects of each subcomponent on WS, (2) indirect effects through PR and PB, (3) the total indirect effect (via PR+PB), and (4) total effects (direct+indirect). Indirect effects were explicitly defined within the structural equation modeling as products of constituent structural paths (eg, DD → PR×PR → WS) using defined parameters in *lavaan*, and were estimated under the same survey-weighted WLSMV framework.

To formally examine whether the effects of DU, DD, and DE differed, Wald chi-square tests were conducted using lavTestWald to compare relevant path coefficients and defined indirect effects across subcomponents. Direct, indirect, and total effects were reported to facilitate the interpretation of heterogeneous mechanisms.

#### Robustness Checks and Supplementary Analyses

To ensure the robustness of our findings against potential methodological artifacts, 2 supplementary analyses were conducted. First, to rigorously test the moderating effect of age without the loss of information associated with dichotomization, we used a survey-weighted generalized linear model (svyglm) to assess the interaction between DL and the full ordinal spectrum of age categories, followed by post hoc simple slope analyses. Std *β* were computed using survey-weighted SDs (std *β*=b×SD[X]/SD[Y]). Second, to address potential selection bias from excluding respondents with “no experience” in the PR variable, we conducted a sensitivity analysis by assigning extreme theoretical values (minimum, midpoint, and maximum) to the missing PR cases within the structural equation modeling framework. All robustness checks are detailed in Multimedia Appendix 2.

### Ethical Considerations

This study was reviewed and approved for exemption by the Institutional Review Board of Gangnam Severance Hospital, Yonsei University (3-2024-0483). This research is a secondary analysis of the KPSDS, a nationwide representative longitudinal dataset provided by the KISDI. The KPSDS is a state-approved statistic (402001). According to the User Guidebook (2023), the original survey was conducted with the informed consent of all participants, and the data were collected in compliance with the Statistics Act of Korea. As the dataset provided to researchers is strictly anonymized and deidentified by KISDI to ensure that no individual participant can be identified, the requirement for obtaining new informed consent for this secondary analysis was waived by the IRB. Regarding compensation, no additional incentives or payments were provided to the participants specifically for this secondary analysis. In the original KPSDS panel survey, participants received standard panel remuneration as determined by KISDI; detailed compensation conditions are not disclosed to secondary data users. Furthermore, we confirm that no individual participants can be identified in any images or supplementary materials associated with this paper.

## Results

### Sample Characteristics

Table 1 presents the demographic characteristics of this study’s sample. The unweighted sample included 4518 Korean internet users aged 15‐69 years, and survey weighting yielded a nationwide representative population of approximately 38.4 million individuals. After weighting, sex distribution became balanced (49.1% female), and age and regional distributions aligned more closely with national population parameters. Educational attainment, occupational status, HI, and perceived SL showed similar patterns before and after weighting.

**Table 1.** Sample characteristics of Korean internet users aged 15‐69 years in a cross-sectional, nationwide representative survey (South Korea, 2023; n=4518). Unweighted and survey-weighted distributions are shown.

Control variable and category	Unweighted (n=4518), n (%)	Weighted (n=38,420,934), n (%)
Sex		
Female	2520 (55.8)	18,858,316 (49.1)
Male	1998 (44.2)	19,562,618 (50.9)
Age group (years)		
Twenties and younger	1072 (23.7)	8,235,437 (21.4)
Thirties	943 (20.9)	6,783,423 (17.7)
Forties	848 (18.8)	7,919,577 (20.6)
Fifties	911 (20.2)	8,350,620 (21.7)
Sixty and older	744 (16.5)	7,131,877 (18.6)
Residential area		
Province	2252 (49.8)	21,309,767 (55.5)
Metropolitan city	1310 (29)	9,768,815 (25.4)
Special city (Seoul)	956 (21.2)	7,342,352 (19.1)
Education level		
High school and below	1709 (37.8)	14,892,087 (38.8)
College	893 (19.8)	7,432,505 (19.3)
University and graduate school	1916 (42.4)	16,096,342 (41.9)
Occupation		
Student and unemployed	511 (11.3)	4,294,116 (11.2)
Employed	3522 (78)	30,259,593 (78.8)
Homemaker	485 (10.7)	3,867,225 (10.1)
Household income (KRW)^a^		
Less than 2 million	150 (3.3)	1,147,170 (3.0)
Less than 4 million	1414 (31.3)	11,123,523 (29.0)
Less than 6 million	1748 (38.7)	15,035,770 (39.1)
6 million and above	1206 (26.7)	11,114,471 (28.9)
Perceived standard of living		
Low	2274 (50.3)	18,565,404 (48.3)
High	2244 (49.7)	19,855,530 (51.7)

a A currency exchange rate of 1 KRW=US $0.00067 was applicable.

### Descriptive Statistics for the Main Variables

Table 2 presents the descriptive statistics for the main variables used in this study, including both unweighted means and SDs and survey-weighted means with SEs. The mean score for WS was 2.903 (SD 1.029) in the unweighted sample and 2.889 (SE 0.018) in the weighted sample.

The mean score for PR was 2.123 (SD 0.641), while PB averaged 3.256 (SD 0.679). The PE had a mean of 3.644 (SD 0.597), MB averaged 3.296 (SD 0.688), and MM was 3.410 (SD 0.615).

Regarding data management perceptions, the mean score for IC was 3.667 (SD 0.623), privacy concern was 3.348 (SD 0.366), interpersonal trust was 3.682 (SD 0.990), and TR was 3.453 (SD 0.628). Weighted means were nearly identical to unweighted estimates.

**Table 2.** Descriptive statistics (unweighted and survey-weighted means and SEs) for main study variables in Korean internet users aged 15‐69 years (South Korea, 2023; n=4518). WS is an ordinal self-reported outcome. For constructs modeled as latent variables in the structural model (eg, PB^a^ and DL^b^), item-averaged composite scores are presented to provide interpretable descriptive statistics on the original Likert scale. DL and its subdimensions were measured on 5-point Likert scales, whereas PR^c^ was measured on a 4-point Likert scale. For PB, descriptive statistics are based on the mean of its observed indicators (q17_8, q17_13, q17_14).

Independent variable	Sample (n=4518), mean (SD)	Weighted (n=38,420,934), mean (SE^d^)
PR	2.123 (0.641)	2.128 (0.011)
PB	3.256 (0.679)	3.259 (0.012)
PE^e^	3.644 (0.597)	3.641 (0.01)
MB^f^	3.296 (0.688)	3.292 (0.011)
MM^g^	3.410 (0.615)	3.403 (0.011)
IC^h^	3.667 (0.623)	3.655 (0.011)
Privacy concern	3.348 (0.366)	3.352 (0.007)
Interpersonal trust	3.682 (0.990)	3.677 (0.017)
TR^i^	3.453 (0.628)	3.464 (0.011)
WS^j^	2.903 (1.029)	2.889 (0.018)
DL	3.329 (0.699)	3.315 (0.014)
DU^k^	3.739 (0.757)	3.716 (0.014)
DD^l^	3.174 (0.779)	3.163 (0.015)
DE^m^	3.187 (0.904)	3.178 (0.017)

a PB: perceived benefit.

b DL: Digital literacy.

c PR: perceived risk.

d Weighted estimates are reported with SEs rather than SDs to reflect the precision of population means under the complex survey design.

e PE: perceived effectiveness of government regulation.

f MB: monetary benefit.

g MM: moral motivation.

h IC: information control.

i TR: institutional trust.

j WS: willingness to share health data.

k DU: digital literacy of use.

l DD: digital literacy of understanding.

m DE: digital literacy of engagement.

### Preliminary Analysis

Before the full-scale structural model analysis, a preliminary analysis was conducted to verify the validity of the data. Upon reviewing the distribution of control variables (Table S15 in Multimedia Appendix 2), sensitivity and selection bias regarding missing value handling (Tables S20 and S21 in Multimedia Appendix 2), assessment of CMV (Tables S22 and S23 in Multimedia Appendix 2), and multicollinearity (Table S25 in Multimedia Appendix 2), no issues were found that violated the model’s main assumptions or caused serious bias. Detailed figures and results are presented in Multimedia Appendix 2.

### Robustness Checks and Supplementary Analyses

#### Age as a Moderator of the Association Between DL and Risk Perception

Figure S1 and Tables S17 and S18 in Multimedia Appendix 2 illustrate the age-specific patterns in the association between DL and PR.

All models were estimated using survey-weighted regression with design-based degrees of freedom.

Table S17 in Multimedia Appendix 2 shows the overall interaction between DL and AG was not statistically significant in the fully adjusted model. However, simple slope analyses revealed heterogeneity in the direction and magnitude of the association across AGs.

Specifically, DL was negatively associated with PR among participants in their thirties (std *β*=−0.093, *P*=.04), whereas positive but nonsignificant associations were observed among participants in their forties (std *β*=0.094, *P*=.08), fifties (std *β*=0.085, *P*=.27), and 60 years and older (std *β*=0.044, *P*=.31). No association was observed among participants in their twenties or younger (std *β*=−0.006, *P*=.91).

These findings suggest potential variation in the relationship between DL and PR across AGs, although the overall interaction was not statistically significant.

#### PR Exclusion Sensitivity Test

Table S20 (Multimedia Appendix 2) showed that under plausible assumptions (exclusion and minimum imputation), the main indirect effects of DD remained consistent in magnitude and statistical significance. In contrast, extreme imputation scenarios (median and maximum) produced unstable estimates, indicating violations of model assumptions. Overall, these findings support the robustness of the main results under realistic assumptions. Additionally, excluded respondents showed significantly lower DL across all subdimensions (all *P*<.001).

#### Measurement Model Evaluation

Confirmatory factor analysis (CFA) was conducted as part of the first structural equation model (model 1), where DL was specified as a second-order latent construct measured by 3 first-order latent constructs: DU, DD, and DE. The model was estimated using a robust diagonally weighted least squares (WLSMV) estimator with survey weights applied.

The robust chi-square test was statistically significant (*χ*^2^_295_=7453.471, *P*<.001), which is expected in large samples. The overall model fit was acceptable: CFI=0.930, TLI=0.923, SRMR=0.052, and robust RMSEA=0.073. All fit indices met conventional criteria (CFI and TLI>0.90, SRMR<0.08, RMSEA<0.08), indicating satisfactory model fit.

#### Indicator Reliability and Internal Consistency

Table 3 presents the comprehensive item-level evaluation of the first-order reflective latent constructs used in model 1. All standardized factor loadings for the indicator items were highly significant (*P*<.001) and demonstrated strong indicator reliability, ranging from 0.600 to 0.867. All item loadings exceeded the conventional threshold of 0.60, confirming that the individual survey items adequately reflect their respective underlying latent constructs.

The internal consistency of the constructs was rigorously assessed using both Cronbach *α* and CR. All constructs exhibited robust reliability, with Cronbach *α* ranging from 0.720 to 0.933, and CR values ranging from 0.776 to 0.954. As all values were well above the recommended minimum threshold of 0.70, the measurement model demonstrated excellent internal consistency.

**Table 3.** Comprehensive measurement model table for model 1.

Construct and number	Mean (SD)	Weighted mean (SE)	Standardized loading	Loading SE	*z* value	*P* value	AVE^a^	CR^b^	α^c^
PB^d^						<.001	0.540	0.776	.720
1	3.29 (0.85)	3.30 (0.01)	0.600	0.020	29.792				
2	3.23 (0.86)	3.24 (0.02)	0.773	0.019	39.892				
3	3.24 (0.83)	3.24 (0.01)	0.813	0.021	39.647				
DU^e^						<.001	0.668	0.923	.894
1	3.80 (0.90)	3.77 (0.02)	0.830	0.008	71.315				
2	3.82 (0.90)	3.80 (0.02)	0.795	0.008	68.873				
3	3.69 (0.96)	3.67 (0.02)	0.818	0.009	68.079				
4	3.73 (0.97)	3.72 (0.02)	0.832	0.009	69.411				
5	3.57 (0.98)	3.54 (0.02)	0.862	0.009	66.289				
6	3.82 (0.91)	3.80 (0.02)	0.762	0.009	62.548				
DD^f^						<.001	0.652	0.954	.933
1	3.17 (1.00)	3.13 (0.02)	0.789	0.010	38.380				
2	3.10 (1.01)	3.07 (0.02)	0.829	0.010	38.315				
3	3.10 (1.02)	3.08 (0.02)	0.837	0.011	37.977				
4	3.09 (1.01)	3.07 (0.02)	0.825	0.010	38.267				
5	3.28 (0.98)	3.28 (0.02)	0.797	0.010	38.692				
6	3.32 (0.96)	3.31 (0.02)	0.799	0.010	37.824				
7	3.29 (0.97)	3.29 (0.02)	0.791	0.010	38.393				
8	3.17 (1.01)	3.16 (0.02)	0.788	0.010	38.464				
9	3.12 (1.03)	3.11 (0.02)	0.797	0.010	38.353				
10	3.13 (1.03)	3.14 (0.02)	0.818	0.010	38.613				
11	3.15 (1.04)	3.16 (0.02)	0.813	0.010	38.280				
DE^g^						<.001	0.708	0.936	.911
1	3.09 (1.13)	3.09 (0.02)	0.832	0.016	16.511				
2	3.23 (1.10)	3.23 (0.02)	0.828	0.016	16.406				
3	3.02 (1.13)	3.00 (0.02)	0.836	0.016	16.406				
4	3.26 (1.06)	3.26 (0.02)	0.850	0.017	16.261				
5	3.25 (1.05)	3.23 (0.02)	0.836	0.016	16.267				
6	3.27 (1.04)	3.26 (0.02)	0.867	0.017	16.362				

a AVE: average variance extracted

b CR: composite reliability.

c α: Cronbach α.

d PB: perceived benefit.

e DU: digital literacy of use.

f DD: digital literacy of understanding.

g DE: digital literacy of engagement.

#### Convergent and Discriminant Validity

Table 3 also shows that convergent validity was supported as the AVE values for all first-order latent constructs surpassed the established benchmark of 0.50, ranging from 0.540 to 0.708. Furthermore, the secondary loadings of DL on its subdimensions were DU=0.683, DD=0.876, and DE=0.949 (all *P*<.001). Although the AVE value (0.298) of the higher-level construct was lower than the threshold of 0.50, this is due to the structural characteristics of the multilevel model. As the AVE of the second-order factor is calculated through the first-order factors, the value tends to be calculated more strictly than in general cases. However, as the factor loadings are high and the CR is sufficiently secured, it can be concluded that the higher-level construct adequately explains the lower-level dimensions.

Discriminant validity was assessed using the Fornell-Larcker criterion and the HTMT ratio. The Fornell-Larcker criterion was satisfied for most pairs of constructs, but between DD and DE, the square root of the AVE of DD (0.808) was lower than the correlation coefficient with DE (model 1: *r*=0.831; model 2: *r*=0.832). To complement these results, the HTMT ratio was additionally examined; the HTMT value between DD and DE was found to be 0.853, and all HTMT values were less than 0.90. Specific results are presented in Tables S6-S11 of Multimedia Appendix 2.

#### Note on Model 2

Model 2 retained the first-order latent constructs (DU, DD, and DE), excluding the higher-order construct (DL). The measurement model remained identical to model 1 without any changes to the specifications of the first-order constructs. Specific details are presented in Tables S9-S11 of Multimedia Appendix 2.

### Structural Model Evaluation (Model 1: H1-H8)

#### Model Fit and Explanatory Power (Model 1)

Model 1 demonstrated satisfactory fit (*χ*²_525_=5372.19, *P*<.001, CFI=0.958, TLI=0.970, RMSEA=0.045, SRMR=0.049). The structural predictors explained 7.3% of the variance in WS (R²_WS_=0.073), 6.2% of PR (R²_PR_=0.062), and 56% of PB (R²_PB_=0.560).

#### Structural Path Analysis and Hypothesis Testing

Hypotheses H1 through H8 were tested using the first structural equation model (model 1), in which DL was modeled as a second-order latent construct composed of 3 first-order latent constructs: DU, DD, and DE.

**Figure 2.**
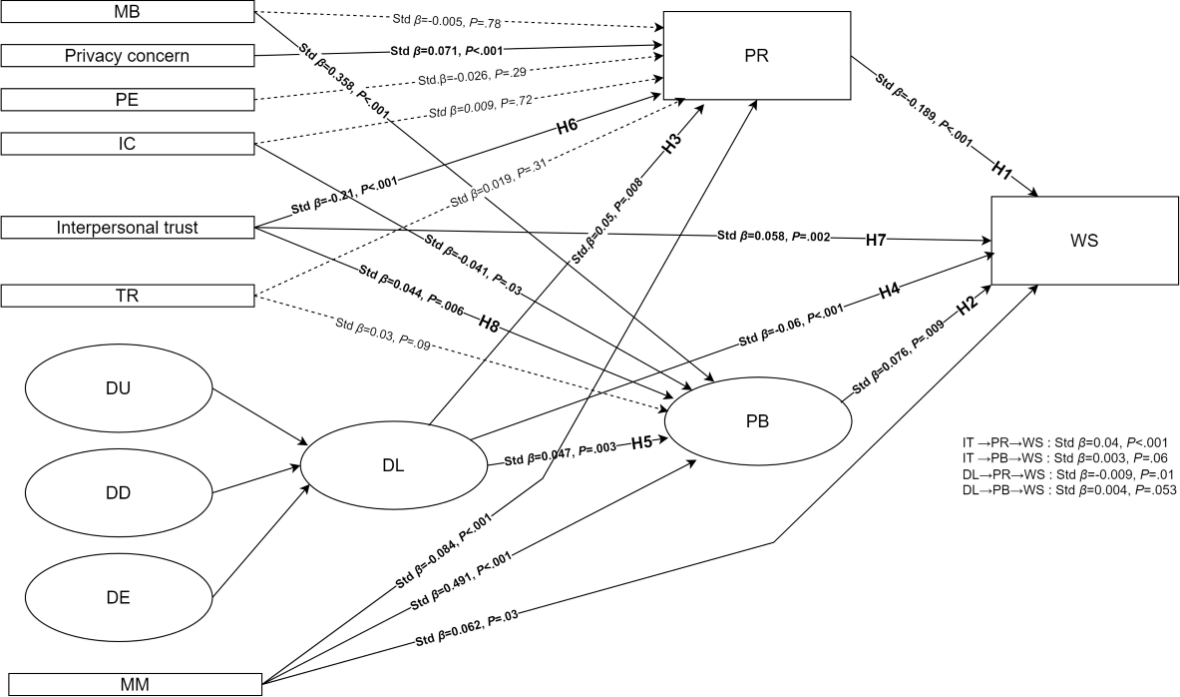
Structural model results (model 1, survey-weighted). Standardized path coefficients (std *β*) from the structural equation modeling testing hypotheses H1-H8 in Korean internet users aged 15-69 years (South Korea, 2023; n=4518). WS was modeled as an ordered outcome. Demographic variables (SX, RA, EL, OC, HI, SL) were analyzed descriptively and via chi-square tests (Table S15, Multimedia Appendix 2) but were not included as covariates in the structural equation modeling. The model focuses on theoretical paths and does not display sociodemographic control variables. Solid lines indicate statistically significant paths, and dashed lines indicate nonsignificant paths. DD: digital literacy of understanding; DE: digital literacy of engagement; DL: digital literacy; DU: digital literacy of use; EL: education level; HI: household income; IC: information control; MB: monetary benefit; MM: moral motivation; OC: occupation; PB: perceived benefit; PE: perceived effectiveness of government regulation; PR: perceived risk; RA: residential area; SL: perceived standard of living; std β: standardized path coefficients; SX: sex; TR: institutional trust; WS: willingness to share health data.

Figure 2 presents the standardized structural path coefficients for model 1.

Regarding WS, PR exhibited the strongest negative direct effect (std *β*=−0.189, *P*<.001). PB showed the strongest positive standardized direct effects (std *β*=0.076, *P*=.009), followed by MM and interpersonal trust (MM: std *β*=0.062, *P*=.03; interpersonal trust: std *β*=0.058, *P*=.002). DL showed a small but statistically significant negative direct association with WS (std *β*=−0.060, *P*<.001).

In predicting PR, interpersonal trust showed the strongest negative effect (std *β*=−0.21, *P*<.001), followed by MM (std *β*=−0.084, *P*<.001). Privacy concern demonstrated a positive standardized effect (std *β*=0.071, *P*<.001), and DL also showed a small but significant positive association (std *β*=0.050, *P*=.008). TR, IC, MB, and PE were not statistically significant predictors of PR (TR: std *β*=0.019, *P*=.31; IC: std *β*=0.009, *P*=.72; MB: std *β*=−0.005, *P*=.78; PE: std *β*=−0.026, *P*=.29).

For PB, MM had the strongest positive standardized effect (std *β*=0.491, *P*<.001), followed by MB (std *β*=0.358, *P*<.001). Interpersonal trust (std *β*=0.044, *P*=.006) and DL (std *β*=0.047, *P*=.003) also showed positive and significant effects. In contrast, IC had a significant negative effect on PB (std *β*=−0.041, *P*=.03), while TR was not a significant predictor (TR: std *β*=0.03, *P*=.09).

Overall, MM was an important predictor of both PB and WS, whereas PR was the main negative predictor of WS. Privacy concern and interpersonal trust were significant antecedents of PR, underscoring the role of psychological and relational factors in health data disclosure.

#### Hypothesis Validation (H1-H8)

H1 through H8 were tested using model 1. As shown in Tables 3 and 4 and Figure 2, a total of 6 of the 8 hypotheses (H1, H2, H5, H6, H7, and H8) were supported, whereas H3 and H4 were not supported.

Regarding WS, PR (H1) had a significant negative effect (std *β*=−0.189, *P*<.001), whereas PB (H2) showed a significant positive effect (std *β*=0.076, *P*=.009). Interpersonal trust (H7) and MM were also positively associated with WS (interpersonal trust: std *β*=0.058, *P*=.002; MM: std *β*=0.062, *P*=.03).

For PR, interpersonal trust (H6) showed a negative effect (std *β*=−0.210, *P*<.001), whereas DL (H3) showed a positive effect (std *β*=0.050, *P*=.008), contrary to the hypothesis.

For PB, DL (H5) and interpersonal trust (H8) both had positive effects (DL: std *β*=0.047, *P*=.003; interpersonal trust: std *β*=0.044, *P*=.006).

Finally, DL (H4) showed a negative direct effect on WS (std *β*=−0.060, *P*<.001), contrary to the hypothesized positive relationship.

**Table 4.** Survey-weighted structural equation modeling results for hypothesized direct paths (H1-H8; model 1) in Korean internet users aged 15‐69 years (South Korea, 2023; n=4518).

Hypothesis	From	To	Estimate	SE	Standard *β*	*z* value	*P* value	Supported
H1: PR^a^ has a negative effect on WS^b^	PR	WS	−0.298	0.025	−.189	−11.820	<.001	✓
H2: PB^c^ has a positive effect on WS	PB	WS	0.122	0.047	.076	2.614	.009	✓
H3: DL has a negative effect on PR	DL^d^	PR	0.057	0.022	.050	2.646	.008	—^e^
H4: DL has a positive effect on WS	DL	WS	−0.109	0.031	−.060	−3.493	<.001	—
H5: DL has a positive effect on PB	DL	PB	0.054	0.018	.047	2.922	.003	✓
H6: interpersonal trust has a negative effect on PR	Interpersonal trust	PR	−0.137	0.011	−.210	−11.960	<.001	✓
H7: interpersonal trust has a positive effect on WS	Interpersonal trust	WS	0.060	0.020	.058	3.044	.002	✓
H8: interpersonal trust has a positive effect on PB	Interpersonal trust	PB	0.028	0.010	.044	2.776	.006	✓

a PR: perceived risk.

b WS: willingness to share health data.

c PB: perceived benefit.

d DL: digital literacy.

e Statistically significant, but the direction of effect is opposite.

### Differential Effects by DL Subcomponent (Model 2: H9)

#### Model Fit and Explanatory Power (Model 2)

To examine whether the subdimensions of DU, DD, and DE differentially influenced privacy-related appraisals and sharing intentions, we estimated a second structural model (model 2).

Model 2 demonstrated satisfactory fit (*χ*²_519_=5255.31, *P*<.001, CFI=0.959, TLI=0.970, RMSEA=0.045, SRMR=0.047). The explanatory power of the model was comparable to model 1, accounting for 7.5% of the variance in willingness to share (R²_WS_=0.075), 9.4% of PR (R²_PR_=0.094), and 57% of PB (R²_PB_=0.570).

#### Global Wald Test Results

As shown in Table 5, the Wald test results for model 2 revealed differences in the direct effects of the DL subfactors (DU, DD, and DE) on sharing intention (WS; *χ*²_2_=7.646, *P*=.02). In other words, this means that the 3 subfactors influence sharing intention in different ways.

**Table 5.** Global Wald test results comparing direct, indirect, and total effects of DL^a^ subdimensions (DU^b^, DD^c^, and DE^d^) on WS^e^ in model 2 (South Korea, 2023; n=4518). Wald chi-square tests examine whether the effects of DL subdimensions (DU, DD, and DE) are equal across pathways (ie, H0: coefficients are equal). Tests are based on survey-weighted structural equation modeling estimates (WLSMV^f^). This table reports omnibus or global Wald tests for equality of effects across DU, DD, and DE. Pairwise Wald comparisons are provided in Table S16 in Multimedia Appendix 2.

Test type	*χ*² (df)		*P* value	Significance
Direct effect (DU/DD/DE → WS)	7.646 (2)		.02	✓
Indirect effect via PR^g^	61.604 (2)		<.001	✓
Indirect effect via PB^h^	5.614 (2)		.06	—^i^
Total indirect effect via PR and PB	56.271 (2)		<.001	✓
Total effects	2.019 (2)		.36	—

a DL: digital literacy.

b DU: digital literacy of use.

c DD: digital literacy of understanding.

d DE: digital literacy of engagement.

e WS: willingness to share health data.

f WLSMV: weighted least squares mean and variance adjusted.

g PR: perceived risk.

h PB: perceived benefit.

i Statistically significant, but the direction of effect is opposite.

The total indirect effect, which combines the indirect effects through PR and PB, also showed differences among the subfactors (*χ*²_2_=56.271, *P*<.001). Breaking this down, there was a difference in the indirect effect through PR (*χ*²_2_=61.604, *P*<.001), but no significant difference in the indirect effect through PB (*χ*²_2_=5.614, *P*=.06).

On the other hand, the total effect, which combines both direct and indirect effects, showed no difference among the subfactors (*χ*²_2_=2.019, *P*=.36). This means that while the processes through which each element exerts influence differ, the magnitude of the final overall impact is similar.

In summary, differences among the subelements of DL were primarily observed in the “path through risk (PR),” while there were no significant differences in the “path through benefit (PB)” or in the overall effect.

The standardized direct, indirect, and total effect estimates for each DL subdimension are presented in Table 6.

**Table 6.** Parameter estimates for direct, indirect, and total effects of DL subdimensions on WS^a^ in model 2 (South Korea, 2023; n=4518). Indirect effects are decomposed via PR^b^ and PB^c^. WS was modeled as an ordered outcome using survey-weighted structural equation modeling (WLSMV^d^ estimation).

Pathway and subdimension	Estimate^e^	SE	Std *β^f^*	*P* value
Direct (DL^g^ → WS)				
DU	−0.028	.033	−.023	.38
DD	−0.141	.043	−.108	.001
DE	.084	.045	.069	.06
Indirect via PR				
DU	−0.035	.007	−.028	<.001
DD	.066	.010	.051	<.001
DE	−0.048	.009	−.039	<.001
Indirect via PB				
DU	−0.004	.003	−.003	.15
DD	.021	.009	.016	.02
DE	−0.012	.006	−.010	.04
Total indirect				
DU	−0.038	.007	−.030	<.001
DD	.087	.013	.066	<.001
DE	−0.060	.011	−.049	<.001
Total effect				
DU	−0.067	.032	−.053	.04
DD	−0.055	.040	−.042	.17
DE	.024	.043	.020	.57

a WS: willingness to share health data.

b PR: perceived risk.

c PB: perceived benefit.

d WLSMV: weighted least squares mean and variance adjusted.

e Estimate: unstandardized coefficient.

f Std *β*: standardized coefficient.

g DL: digital literacy.

#### Differential Direct and Indirect Effects

**Figure 3.**
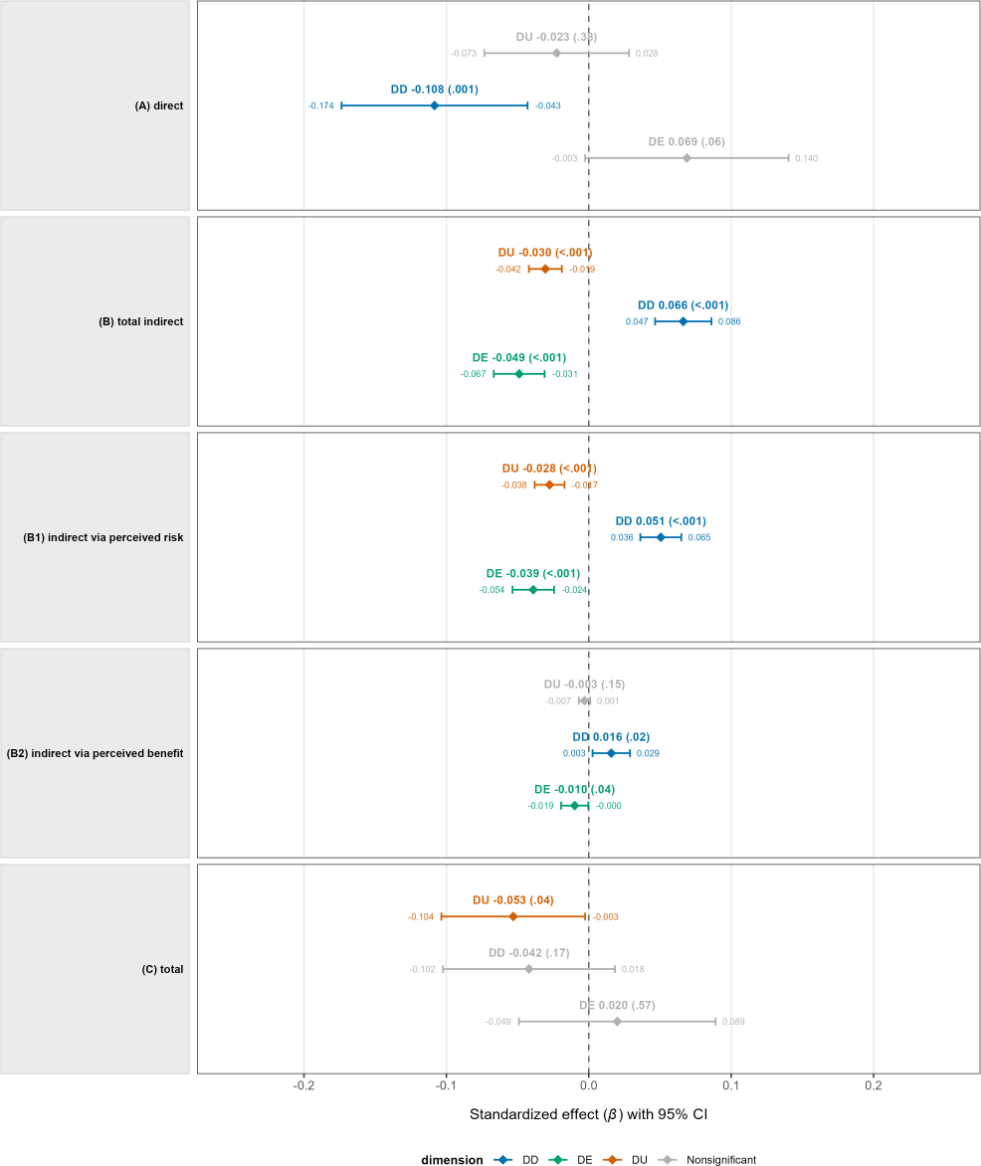
Standardized direct, indirect, and total effects of DL subdimensions on willingness to share health data (model 2). Effects are presented as standardized coefficients (std *β*) with 95% CIs. Panels show (A) direct effects, (B) total indirect effects, (B1) indirect effects via perceived risk, (B2) indirect effects via perceived benefit, and (C) total effects. DL subdimensions include DU, DD, and DE. Panel (B) presents the total indirect effects, which are decomposed into indirect effects via perceived risk (B1) and perceived benefit (B2). DD: digital literacy of understanding; DE: digital literacy of engagement; DU: digital literacy of use.

Figure 3 shows the standardized direct, indirect, and total effects of DL subdimensions (DU, DD, and DE) on health data sharing intention (WS).

A significant negative effect was observed only for DD (std *β*=−0.108, *P*=.001), while DU (std *β*=−0.023, *P*=.38) and DE (std *β*=0.069, *P*=.06) were not statistically significant.

In terms of total indirect effects considering both PR and PB, DD showed a significant positive effect (std *β*=0.066, *P*<.001), while DU and DE showed significant negative effects (DU: std *β*=−0.030, *P*<.001; DE: std *β*=−0.049, *P*<.001).

Upon decomposition, for the PR mediation path, DD showed a significant positive indirect effect (std *β*=0.051, *P*<.001), whereas DU and DE showed significant negative indirect effects (DU: std *β*=*−*0.028, *P*<.001; DE: std *β*=−0.039, *P*<.001), reflecting the opposite direction of the DL → PR path and the PR → WS path.

For the PB mediation path, DD showed a significant positive indirect effect (std *β*=0.016, *P*=.02), while DE showed a small but statistically significant negative indirect effect (std *β*=−0.010, *P*=.04). DU also showed a negative indirect effect through PB (std *β*=−0.003, *P*=.15), but this was not statistically significant. Looking at the overall effects, only DU showed a statistically significant negative effect on WS (std *β*=−0.053, *P*=.04). On the other hand, DD (std *β*=−0.042, *P*=.17) and DE (std *β*=0.020, *P*=.57) were not statistically significant, and the CI includes 0, indicating uncertainty regarding the direction of the effect.

## Discussion

### Principal Results

This study analyzed the factors influencing an individual’s WS based on CPM theory [15,30] and privacy calculus theory [19,24,36]. In particular, it examined the impact of psychological and social factors, such as DL, interpersonal trust, and MM, on data sharing behavior.

The results supported the core assumptions of privacy calculus theory. Specifically, the WS is determined by the balance between PR and PB, with PB appearing as the strongest positive predictor. Additionally, MM (std *β*=0.062, *P*=.03) and interpersonal trust (std *β*=0.058, *P*=.002) also showed significant positive effects, suggesting that data sharing decisions are formed not only by personal gain but also by altruistic values and relational factors [26,29].

Among the components of DL, “understanding” was found to have the greatest indirect effect through PR and PB. Contrary to existing hypotheses, DL was found to both increase PR (std *β*=0.050, *P*=.008) and directly reduce willingness to share (std *β*=−0.060, *P*<.001).

This suggests that an individual’s cognitive ability indirectly shapes data sharing behavior by structuring evaluations of risk and benefit, and that DL can act as a kind of “filter” in the data sharing decision-making process, leading to more prudent judgment.

These results highlight the important role of user competence in shaping health data sharing behavior [32] and are consistent with the CPM theory [8,35], which posits that data sharing is a trust-based boundary management process. In other words, individuals manage information through privacy boundaries that change according to digital competence and social context, rather than fixed standards, and DL and interpersonal trust play a key role in this boundary-setting process [15,30]. In this regard, this study complements the privacy calculus perspective and demonstrates that data sharing is not merely a rational choice but a dynamic process involving cognitive evaluation and social factors.

### Age Interaction

The results of this study suggest that the relationship between DL and PR may be moderated by age. While no significant association between the 2 variables was observed in the overall sample, post hoc analysis confirmed a statistically significant relationship between DL and PR (std *β*=−0.093, *P*=.04) only in the thirties AG. Although statistical significance was not reached in other AGs, it is noteworthy that the slopes varied by age.

These results suggest that DL may be reflected differently in how risk is assessed depending on age. In particular, considering that DL includes an understanding of privacy protection policies and the context of data usage, it is possible that for a specific AG (thirties), these competencies served as a criterion for evaluating privacy risk.

This partially aligns with the CPM theory, which posits that an individual’s privacy boundary setting varies depending on the context of information and the individual’s rules. However, as these results are based on post hoc analysis, a cautious approach is required when generalizing them to all AGs or interpreting them as definitive conclusions; further verification is necessary through follow-up research that considers generational characteristics.

### Comparison With Prior Work

The results of this study are largely consistent with prior research based on privacy calculus. Previous studies have shown that WS decreases as PR increases [3,5,45] and increases as PBs increase [4,6,25]. However, unlike earlier studies suggesting that DL reduces risk through greater technological control, the present findings align with more recent research indicating that DL may increase risk sensitivity [46]. This provides a possible explanation for why hypotheses H3 and H4 were not supported as expected. DL may enable individuals to recognize subtle threats in increasingly complex data environments, which can lead to more cautious data-sharing behavior. This tendency toward greater caution associated with higher DL offers a new perspective on traditional privacy calculus models. This relationship may be particularly important in the context of personal health data (PGHD), which is often sensitive and frequently collected.

The finding that MM has a significant influence on WS is consistent with prior research [26,29]. While previous studies have primarily focused on individual cost-benefit analysis, the present study highlights the importance of collective values, such as contributing to public health.

The finding that interpersonal trust has a significant positive effect on willingness to share is also consistent with prior research [8,32,35,47]. However, while interpersonal trust was significant, variables reflecting institutional factors (eg, perceived institutional effectiveness, PE) were not. This partially contrasts with previous studies suggesting that it can reduce PR [48]. This difference may reflect variation in how different types of trust influence data-sharing decisions. In particular, in cultures such as Korea that emphasize personal relationships and social networks, interpersonal trust may play a more prominent role than formal TR, which may help explain differences from findings in Western contexts [47,49]. In addition, institutional factors in this study were operationalized as perceived institutional effectiveness rather than trust in government, which may also contribute to these differences.

Finally, this study used nationwide representative data from Korean internet users rather than specific subpopulations, thereby enhancing the generalizability of the findings and strengthening their policy relevance compared to prior research [4,5,14].

### Implications

To promote health data sharing within digital health systems, policy and intervention strategies should extend beyond technical infrastructure to incorporate psychological and social dimensions. Our analysis identified the “understanding” component of DL as having the strongest indirect effect on WS. This suggests that DL education should emphasize critical thinking, ethical reasoning, and data interpretation rather than focusing solely on technical or operational skills [33,34].

Although the overall interaction effect between age and DL did not reach statistical significance (*P*=.06), the heterogeneity of the slopes by AG observed in the post hoc analysis offers notable implications. The positive association observed in some AGs suggests the possibility that high literacy may have both facilitating and inhibiting effects, potentially increasing awareness of data-related risks and making people cautious about sharing. Therefore, when establishing future strategies to promote the sharing of personal health data (PGHD), it should move beyond uniform digital competency training and simultaneously implement trust-based governance and specific protective measures that consider age-specific characteristics.

Additionally, the variability in willingness to share PGHD depending on its intended use highlights the importance of contextual sensitivity. While participants expressed high willingness to share data for public health or research purposes, their willingness decreased markedly when the purpose involved commercial use, largely due to elevated perceptions of risk [9,14,50]. These findings indicate the need for purpose-specific consent models and clearer communication strategies that reflect the intended use of health data.

### Policy Recommendations

The findings of this study yield the following policy implications for advancing digital health strategies and health data governance frameworks.

First, consent mechanisms should reflect the sensitivity of data types. PGHD range from relatively low-risk information (eg, step count or sleep patterns) to highly sensitive data (eg, heart rate, GPS, or stress levels). A one-size-fits-all consent process is inadequate. Instead, category-specific notifications and granular, opt-in consent designs—particularly for potential commercial uses—are essential for respecting user preferences and mitigating PRs [9,47,50].

Second, data-sharing frameworks should emphasize public value. Given the strong effect of MM on WS, data initiatives should be framed around collective benefits, such as medical research and disease prevention [26,29]. Institutions that collect or use PGHD should clearly communicate how data use aligns with public interest to increase transparency and trust.

Third, DL should be a core focus of policy. The “understanding” component of DL showed the most significant indirect effect on willingness to share. This underscores the need for education that fosters critical judgment, ethical awareness, and user agency, rather than focusing solely on technical skills [33,34,51]. Literacy strategies should move toward participatory and contextual learning that enables individuals to make informed decisions in digital health environments.

Fourth, trust-centered data governance must be prioritized. For PGHD to be accepted and effectively used—whether by governments, health care institutions, or corporations—technical safeguards must be supplemented with transparent practices, clear accountability mechanisms, and meaningful stakeholder engagement. Interpersonal trust plays a critical role in shaping users’ perceptions and behaviors, and governance models must reflect this social dimension [8,32,35].

Taken together, these strategies can foster a sustainable data-sharing culture grounded in public interest, ethical responsibility, and user empowerment.

### Limitations

This study has several limitations. First, the cross-sectional, self-reported design limits causal inference and may be subject to CMV. Although Harman single-factor test suggested that a single factor did not dominate the covariance among measures (including a polychoric-based variant), CMV cannot be fully ruled out. Second, DL was measured using a general media literacy scale rather than a health-specific instrument such as eHEALS [16,17], reflecting the broader, nonclinical digital environments in which PGHD are created and shared. However, this may limit comparability with prior studies that operationalized DL using eHEALS. Third, WS was assessed using a single self-reported item that did not specify the data recipient (eg, government agencies, academic researchers, or commercial vendors). As sharing intentions is known to vary by target, this ambiguity may weaken construct validity and blur distinctions between institutional and interpersonal trust. Fourth, although attitudes differed by data use purpose (eg, public vs commercial), no behavioral or scenario-based validations were conducted. In addition, respondents who reported “no experience” across all PR items were excluded because PR could not be computed, which may slightly limit generalizability to internet users with minimal exposure to the relevant digital context; however, the excluded proportion was small (1.38%). Future research should use longitudinal, qualitative, or scenario-based experimental designs to validate these mechanisms and better capture behavioral outcomes. The AVE of the meta-DL construct was found to be lower than the recommended threshold (AVE=0.298). However, the CR was 0.879, satisfying the general criterion (CR≥0.70). While low AVE values are common in hierarchical second-factor models due to the integration of various subdimensions, this suggests the possibility that convergent validity may be limited at the higher level. Therefore, caution is required in interpreting the meta-construct. Finally, the model’s relatively low explanatory power suggests that a significant portion of the variability in the WS remains unexplained. This appears to reflect the complex real-world decision-making processes occurring within heterogeneous populations. Nevertheless, this study contributes to understanding the mechanisms related to DL and privacy awareness. Future research should include additional contextual factors to enhance explanatory power.

Despite these limitations, this study offers several notable strengths. First, unlike previous research limited to specific patient groups or technology users [4,14,21], this study analyzed nationwide representative data from general internet users in Korea, thereby enhancing generalizability. Second, by disaggregating DL into 3 subdimensions—use, understanding, and engagement—it provides practical insights for designing targeted digital health education policies [34,37,51]. Third, the integration of both privacy calculus and CPM theories enabled a multidimensional analysis of data-sharing behavior, incorporating both rational decision-making and privacy boundary management [15,30].

### Conclusions

This study identified key psychological and social factors influencing the WS based on privacy calculus and CPM theories. The results showed that PBs had the strongest positive influence, while PRs had the strongest negative influence on willingness to share. Additionally, MM, interpersonal trust, and the “understanding” dimension of DL were significant factors, indicating that health data sharing involves both cognitive and social processes beyond simple cost-benefit evaluations.

Considering that the relationship between DL and risk perception varies by age, tailored policies and educational interventions are needed. Furthermore, as WS differs depending on the purpose (eg, public vs commercial) and sensitivity, providing clear information and purpose-specific consent mechanisms is essential. These findings highlight the importance of trust-based, user-centric strategies to promote health data sharing and support the expansion of digital health care.

Future research should improve measurement tools to reduce interpretation bias and better capture contextual factors influencing health data sharing. In particular, health literacy instruments such as eHEALS may allow more precise assessment of digital capabilities. Additionally, studies involving specific clinical populations are needed to examine how willingness to share data translates into actual health behaviors and outcomes.

## Supplementary material

10.2196/75448 Multimedia Appendix 1 Variable definitions and questionnaire items used in this study.

10.2196/75448 Multimedia Appendix 2 Data tables and figures.

## Abbreviations

AG: age group
AVE: average variance extracted
CFI: comparative fit index
CHERRIES: Checklist for Reporting Results of Internet E-Surveys
CMV: common method variance
CPM: communication privacy management
CR: composite reliability
DD: digital literacy of understanding
DE: digital literacy of engagement
DL: digital literacy
DU: digital literacy of use
DWLS: Diagonally Weighted Least Squares
eHEALS: eHealth Literacy Scale
HI: household income
HTMT: heterotrait-monotrait
IC: information control
KISDI: Korea Information Society Development Institute
KPSDS: Korea Panel Survey on the Digital Society
MB: monetary benefit
MM: moral motivation
PB: perceived benefit
PE: perceived effectiveness of government regulation
PGHD: patient-generated health data
PR: perceived risk
RA: residential area
RMSEA: root mean square error of approximation
SL: perceived standard of living
SRMR: standardized root mean square residual
std *β*: standardized coefficient
std all: Fully standardized estimates
TLI: Tucker-Lewis index
TR: institutional trust
WLSMV: weighted least squares mean and variance adjusted
WS: willingness to share health data
