# Correction: Digital Literacy and Interpersonal Trust as Predictors of Willingness to Share Patient-Generated Health Data Among Korean Internet Users: Cross-Sectional Study Using Privacy Calculus and Communication Privacy Management Theories

**DOI:** 10.2196/103310

**Published:** 2026-07-31

**Authors:** Dongsu Lee, Wonseuk Jang

**Affiliations:** 1 Future Medicine Research Center, Gangnam Severance Hospital, College of Medicine, Yonsei University, Seoul, Republic of Korea; 2 Department of Medical Device Engineering and Management, Yonsei University College of Medicine, 50-1, Yonsei-Ro, Seodaemun-gu, Seoul, 03722, Republic of Korea, 82-2-2019-5442

In “Digital Literacy and Interpersonal Trust as Predictors of Willingness to Share Patient-Generated Health Data Among Korean Internet Users: Cross-Sectional Study Using Privacy Calculus and Communication Privacy Management Theories” [1], the authors noted one error.

The affiliation of the corresponding author has been corrected. The following affiliation was revised:

Future Medicine Research Center, Gangnam Severance Hospital

College of Medicine

Yonsei University

20 63rd Road

Unju-ro, Gangnam-gu

Seoul, 06229

Republic of Korea

The new information was added as affiliation 2, and reads:

Department of Medical Device Engineering and Management

Yonsei University College of Medicine

50-1, Yonsei-Ro, Seodaemun-gu

Seoul, 03722

Republic of Korea

The correction will appear in the online version of the paper on the JMIR Publications website, together with the publication of this correction notice. Because this was made after submission to PubMed, PubMed Central, and other full-text repositories, the corrected article has also been resubmitted to those repositories.
