# Supplementary material for: Digital Literacy and Interpersonal Trust as Predictors of Willingness to Share Patient-Generated Health Data Among Korean Internet Users: Cross-Sectional Study Using Privacy Calculus and Communication Privacy Management Theories
Source: J Med Internet Res. 2026 May 27;28:e75448. doi: 10.2196/75448 (PMC13215976; doi:10.2196/75448)
Supplement: Multimedia Appendix 2 [file jmir-v28-e75448-s002.docx]

This multimedia appendix provides supplementary materials supporting the main analyses, including measurement model evaluation, discriminant validity assessment, structural results, survey-weighted analyses, age interaction models, PR sensitivity analyses, and selection bias checks.

**Abbreviations**

- PB: Perceived benefit
- DU: Digital literacy of use
- DD: Digital literacy of understanding
- DE: Digital literacy of engagement
- DL: Digital literacy

## **Section S1.** Measurement model evaluation for Model 1 and Model 2 (CFA)

### Measurement summary for latent constructs (Model 1)

**Table S1.** Measurement properties of latent constructs in Model 1 (survey-weighted confirmatory factor analysis). This table summarizes the measurement properties of first-order and second-order latent constructs, including factor loading ranges, average variance extracted (AVE), composite reliability (CR), and Cronbach α. All estimates are based on survey-weighted CFA using WLSMV estimation.

| Construct | No. of items | Loading range | AVE | CR | α |
| --- | --- | --- | --- | --- | --- |
| PB | 3 | 0.600–0.813 | 0.540 | 0.776 | 0.720 |
| DU | 6 | 0.762–0.862 | 0.668 | 0.923 | 0.894 |
| DD | 11 | 0.788–0.837 | 0.652 | 0.954 | 0.933 |
| DE | 6 | 0.828–0.867 | 0.708 | 0.936 | 0.911 |
| DL 2nd-order | 23 | 0.683–0.949 | 0.298 | 0.879 | 0.952 |

Note: AVE: average variance extracted; CR: composite reliability; α: Cronbach alpha.

Acceptable thresholds: AVE ≥ .50, CR ≥ .70.

### Second-order digital literacy measurement details (Model 1)

**Table S2.** Second-order measurement model of digital literacy showing standardized loadings of first-order dimensions (Model 1). Standardized factor loadings, standard errors, and z-values are reported for the higher-order construct digital literacy (DL) and its first-order dimensions: use (DU), understanding (DD), and engagement (DE).

| Construct | Dimension | Standardized loading | Loading SE | z-value | P value |
| --- | --- | --- | --- | --- | --- |
| DL 2nd-order | DU | 0.683 | 0.027 | 34.228 | P < .001 |
|  | DD | 0.876 | 0.064 | 28.248 | P < .001 |
|  | DE | 0.949 | 0.205 | 14.603 | P < .001 |

Note DU: digital literacy of use; DD: digital literacy of understanding; DE: digital literacy of engagement.

### Measurement model fit indices (Model 1)

**Table S3.** Model fit indices for the measurement model (Model 1, survey-weighted WLSMV estimation). Model fit was evaluated using robust (scaled) χ², CFI, TLI, RMSEA, and SRMR. Values meet commonly recommended thresholds for acceptable fit in SEM.

| Measure | Value |
| --- | --- |
| χ² (scaled) | 7,453.471 |
| Degrees of freedom | 295 |
| P value (scaled) | < .001 |
| CFI (scaled) | 0.930 |
| TLI (scaled) | 0.923 |
| RMSEA (scaled) | 0.073 |
| SRMR | 0.052 |

Note

CFI: comparative fit index; TLI: Tucker–Lewis index; RMSEA: root mean square error of approximation; SRMR: standardized root mean square residual.

Acceptable thresholds: CFI/TLI ≥ .90, RMSEA ≤ .08, SRMR ≤ .08.

### Measurement summary for latent constructs (Model 2)

**Table S4.** Measurement properties of latent constructs in Model 2 (survey-weighted confirmatory factor analysis). This table reports reliability and convergent validity indices for latent constructs retained in Model 2, where digital literacy subdimensions are modeled separately.

| Construct | No. of items | Loading range | AVE | CR | α |
| --- | --- | --- | --- | --- | --- |
| PB | 3 | 0.601–0.816 | 0.540 | 0.776 | 0.720 |
| DU | 6 | 0.762–0.861 | 0.668 | 0.923 | 0.894 |
| DD | 11 | 0.788–0.837 | 0.652 | 0.954 | 0.933 |
| DE | 6 | 0.828–0.868 | 0.708 | 0.936 | 0.911 |

Note: AVE: average variance extracted; CR: composite reliability; α: Cronbach alpha.

Acceptable thresholds: AVE ≥ .50, CR ≥ .70.

### Measurement model fit indices (Model 2)

**Table S5.** Model fit indices for the measurement model (Model 2, survey-weighted WLSMV estimation).

| Measure | Value |
| --- | --- |
| χ² (scaled) | 7,676.402 |
| Degrees of freedom | 293 |
| P value (scaled) | < .001 |
| CFI (scaled) | 0.928 |
| TLI (scaled) | 0.920 |
| RMSEA (scaled) | 0.075 |
| SRMR | 0.050 |

Note: Same interpretation criteria as Table S3.

## **Section S2.** Discriminant validity assessment (CFA)

### Fornell–Larcker matrix for Model 1

**Table S6.** Fornell–Larcker discriminant validity matrix for latent constructs (Model 1, survey-weighted correlations). Diagonal elements represent the square root of AVE. Discriminant validity is supported when each diagonal value exceeds the corresponding inter-construct correlations.

| Construct | PB | DU | DD | DE |
| --- | --- | --- | --- | --- |
| PB | **0.735** | 0.159 | 0.204 | 0.221 |
| DU | 0.159 | **0.817** | 0.598 | 0.648 |
| DD | 0.204 | 0.598 | **0.808** | 0.831 |
| DE | 0.221 | 0.648 | 0.831 | **0.842** |

Note: Diagonal elements represent $\sqrt{\mathrm{AVE}}$. Discriminant validity is supported when $\sqrt{\mathrm{AVE}}$exceeds inter-construct correlations.

### Heterotrait–monotrait (HTMT) for Model1

**Table S7.** Heterotrait–monotrait (HTMT) ratio matrix for latent constructs (Model 1, unweighted polychoric correlations). HTMT ratios were computed from the unweighted polychoric correlation matrix as a supplementary assessment of discriminant validity.

| Construct | PB | DU | DD | DE |
| --- | --- | --- | --- | --- |
| PB |  |  |  |  |
| DU | 0.223 |  |  |  |
| DD | 0.213 | 0.558 |  |  |
| DE | 0.140 | 0.612 | 0.853 |  |

Note: HTMT values below .85 (strict) or .90 (liberal) indicate adequate discriminant validity.

### HTMT threshold evaluation (Model 1)

**Table S8.** Evaluation of HTMT ratios against threshold criteria (.85 and .90) (Model 1).

| Construct | Max HTMT | <.85 | <.90 |
| --- | --- | --- | --- |
| PB | 0.223 | Yes | Yes |
| DU | 0.612 | Yes | Yes |
| DD | 0.853 | No | Yes |
| DE | 0.853 | No | Yes |

Note: “Yes” indicates that the HTMT value is below the specified threshold.

### Full Fornell–Larcker matrix (Model 2)

**Table S9.** Fornell–Larcker discriminant validity matrix (Model 2, survey-weighted correlations).

| Construct | PB | DU | DD | DE |
| --- | --- | --- | --- | --- |
| PB | 0.735 | 0.214 | 0.228 | 0.148 |
| DU | 0.214 | 0.817 | 0.594 | 0.652 |
| DD | 0.228 | 0.594 | 0.808 | 0.832 |
| DE | 0.148 | 0.652 | 0.832 | 0.842 |

Note: Interpretation is identical to Table S6.

### HTMT matrix (Model 2)

**Table S10.** HTMT ratio matrix (Model 2, unweighted polychoric correlations).

| Construct | PB | DU | DD | DE |
| --- | --- | --- | --- | --- |
| PB |  |  |  |  |
| DU | 0.223 |  |  |  |
| DD | 0.213 | 0.558 |  |  |
| DE | 0.140 | 0.612 | 0.853 |  |

Note: Interpretation is identical to Table S7.

### HTMT threshold evaluation (Model 2)

**Table S11.** Evaluation of HTMT ratios against threshold criteria (.85 and .90) (Model 2).

| Construct | Max HTMT | <.85 | <.90 |
| --- | --- | --- | --- |
| PB | 0.223 | Yes | Yes |
| DU | 0.612 | Yes | Yes |
| DD | 0.853 | No | Yes |
| DE | 0.853 | No | Yes |

Note: Interpretation is identical to Table S8.

# **Section S3.** Structural results

### Structural model fit indices for Model 1 and Model 2 (weighted)

**Table S12.** Global fit indices for structural equation models (Model 1 and Model 2, survey-weighted WLSMV estimation).

| Model | χ² (Scaled) | df | P value | CFI | TLI | RMSEA | SRMR |
| --- | --- | --- | --- | --- | --- | --- | --- |
| Model 1 (H1-H8) | 5372.19 | 525 | <.001 | 0.958 | 0.970 | 0.045 | 0.049 |
| Model 2 (H9) | 5255.31 | 519 | <.001 | 0.959 | 0.970 | 0.045 | 0.047 |

### Explanatory power of structural models

**Table S13.** Explained variance (R²) of endogenous variables in structural models (survey-weighted).

| Variable | Model 1 | Model 2 |
| --- | --- | --- |
| WS | 0.073 | 0.075 |
| PR | 0.062 | 0.094 |
| PB | 0.560 | 0.570 |
| DU | 0.484 |  |
| DD | 0.746 |  |
| DE | 0.916 |  |

### Standardized effects (wide format)

**Table S14.** Standardized direct, indirect, and total effects of digital literacy subdimensions (wide-format summary, Model 2). Standardized coefficients (Std.β) are presented with corresponding P values for direct, indirect (via PR and PB), and total effects.

| Pathway | DU | DD | DE |
| --- | --- | --- | --- |
| Direct effects (DU/DD/DE → WS) | -0.023 (P = .38) | -0.108 (P = .001) | 0.069 (P = .06) |
| Indirect effects via PR | -0.028 (P < .001) | 0.051 (P < .001) | -0.039 (P < .001) |
| Indirect effects via PB | -0.003 (P = .15) | 0.016 (P = .02) | -0.010 (P = .04) |
| Total indirect effects via PR and PB | -0.030 (P < .001) | 0.066 (P < .001) | -0.049 (P < .001) |
| Total effects | -0.053 (P = .04) | -0.042 (P = .17) | 0.020 (P = .57) |

Note: Effect means Std.β. Includes both direct and mediated effects.

### Survey-weighted chi-square tests (socio-demographics vs willingness to share health data)

**Table S15.** Survey-weighted chi-square tests of associations between socio-demographic characteristics and willingness to share health data.

| variable | χ² | df | P value | significance |
| --- | --- | --- | --- | --- |
| sex | 0.821 | 4 | .51 | ✗ |
| age group | 1.293 | 16 | .19 | ✗ |
| residential area | 5.398 | 8 | <.001 | ✓ |
| education level | 1.324 | 8 | .23 | ✗ |
| occupation | 1.293 | 8 | .24 | ✗ |
| household income | 3.416 | 11 | <.001 | ✓ |
| perceived standard of living | 4.667 | 4 | <.001 | ✓ |

Note: Weighted design reflects nationally representative estimates.

### Wald test result for subcomponents (weighted model 2)

**Table S16.** Wald tests comparing the effects of digital literacy subdimensions (DU, DD, DE) in Model 2.

| Test type | Comparison | χ² | df | P value | significance |
| --- | --- | --- | --- | --- | --- |
| Direct effect (DU/DD/DE → WS) | DU vs DD | 3.919 | 1 | .048 | ✓ |
|  | DU vs DE | 3.002 | 1 | .08 | ✗ |
|  | DD vs DE | 7.511 | 1 | .006 | ✓ |
| Indirect effects via PB | DU vs DD | 5.606 | 1 | .02 | ✓ |
|  | DU vs DE | 1.977 | 1 | .16 | ✗ |
|  | DD vs DE | 5.094 | 1 | .02 | ✓ |
| Indirect effects via PR | DU vs DD | 60.569 | 1 | <.001 | ✓ |
|  | DU vs DE | 1.172 | 1 | .28 | ✗ |
|  | DD vs DE | 41.122 | 1 | <.001 | ✓ |
| Total effects | DU vs DD | 0.051 | 1 | .82 | ✗ |
|  | DU vs DE | 2.019 | 1 | .16 | ✗ |
|  | DD vs DE | 1.048 | 1 | .31 | ✗ |

# **Section S4.** Additional robustness and interaction analyses

### Ordinal interaction model results

**Table S17.** Wald test for the interaction between digital literacy and ordinal age in predicting perceived risk (survey-weighted model).

| Test | Type | Statistic | df | P value |
| --- | --- | --- | --- | --- |
| DL × ordered age spectrum | Wald χ² | 2.281 | 4 | .06 |

Note: Age treated as an ordinal variable.

### Categorical interaction model results

**Table S18.** Results of categorical interaction model (digital literacy × age group) predicting perceived risk.

| term | estimate | SE | t | P value | Std.β |
| --- | --- | --- | --- | --- | --- |
| (Intercept) | 2.593 | 0.188 | 13.794 | < .001 |  |
| Digital Literacy | -0.005 | 0.044 | -0.113 | .91 | -0.006 |
| Age 30s | 0.355 | 0.209 | 1.7 | .09 |  |
| Age 40s | -0.284 | 0.222 | -1.277 | .20 |  |
| Age 50s | -0.237 | 0.272 | -0.87 | .38 |  |
| Age 60 and above | -0.181 | 0.186 | -0.972 | .33 |  |
| IT | -0.135 | 0.011 | -12.558 | < .001 | -0.207 |
| PE | -0.023 | 0.023 | -0.981 | .33 | -0.021 |
| MB | 0.002 | 0.018 | 0.104 | .92 | 0.002 |
| PC | 0.114 | 0.034 | 3.349 | < .001 | 0.066 |
| IC | 0.01 | 0.023 | 0.424 | .67 | 0.009 |
| MM | -0.089 | 0.023 | -3.934 | < .001 | -0.085 |
| Digital Literacy x Age 30s | -0.078 | 0.06 | -1.304 | .19 |  |
| Digital Literacy x Age 40s | 0.089 | 0.065 | 1.367 | .17 |  |
| Digital Literacy x Age 50s | 0.081 | 0.081 | 1.008 | .31 |  |
| Digital Literacy x Age 60 and above | 0.044 | 0.058 | 0.754 | .45 |  |

Note: Age categorized into discrete groups.

### Simple slope analysis (post-hoc)

**Table S19.** Simple slope analysis of digital literacy predicting perceived risk across age groups.

| Age group | Slope DL to PR | SE | t | Std.β | P value |
| --- | --- | --- | --- | --- | --- |
| 20s and below | -0.005 | 0.044 | -0.113 | -0.006 | .91 |
| 30s | -0.083 | 0.041 | -2.011 | -0.093 | .04 |
| 40s | 0.084 | 0.049 | 1.723 | 0.094 | .08 |
| 50s | 0.076 | 0.069 | 1.109 | 0.085 | .27 |
| 60 and above | 0.039 | 0.039 | 1.013 | 0.044 | .31 |

Note: Simple slopes estimated at representative levels of age.

### PR sensitivity analysis results

**Table S20.** Sensitivity analysis under alternative specifications of perceived risk (PR) in Model 2 (South Korea, 2023; n_analytic = 4,518, n_sensitivity = 4,581).

| Scenario | Path | Std.β | P value |
| --- | --- | --- | --- |
| 1. Main (Exclude) | DD → PR → WS | 0.051 | <.001 |
|  | DD → PB → WS | 0.016 | .02 |
|  | DD total effect | -0.042 | .17 |
| 2. PR = 1 (Min) | DD → PR → WS | 0.046 | <.001 |
|  | DD → PB → WS | 0.018 | .01 |
|  | DD total effect | -0.04 | .20 |
| 3. PR = 2.5 (Mid) | DD → PR → WS | 1.032 | <.001 |
|  | DD → PB → WS | 0.263 | <.001 |
|  | DD total effect | -0.114 | .01 |
| 4. PR = 4 (Max) | DD → PR → WS | 1.257 | <.001 |
|  | DD → PB → WS | 0.351 | <.001 |
|  | DD total effect | -0.122 | .02 |

Note: Results support the robustness of the key DD indirect effect under the main exclusion and minimum-imputation scenarios. Median and maximum imputations are presented as extreme sensitivity scenarios and should be interpreted cautiously because they produced unstable estimates. Standardized indirect and total effects are reported for the DD subcomponent across alternative specifications. P values are reported as P < .001 when applicable; otherwise, exact P values are reported (no leading zero). Rows correspond to sensitivity specifications assessing the robustness of the indirect and total effects when imputing values (min = 1, mid = 2.5, max = 4) for the 63 respondents excluded from the main analysis due to missing perceived risk.

### Selection bias check for PR exclusion

**Table S21.** Comparison of included and excluded samples to assess selection bias due to missing perceived risk.

| Variable | Mean (Included) | Mean (Excluded) | t | P value |
| --- | --- | --- | --- | --- |
| DL | 3.31 | 2.03 | -8.213 | <.001 |
| DU | 3.72 | 2.17 | -9.029 | <.001 |
| DD | 3.16 | 2.02 | -7.262 | <.001 |
| DE | 3.18 | 1.91 | -8.475 | <.001 |

Note: Although statistically significant differences were observed between included and excluded groups, the excluded group represented a small proportion of the sample. Sensitivity analyses suggested that the impact of this difference on the main results was limited.

### CMV test (4, 5 score)

**Table S22.** Common method variance (CMV) test results for 4-point scale variables.

| n_complete | p_raw | p_poly |
| --- | --- | --- |
| 2284 | 0.405 | 0.460 |

**Table S23.** Common method variance (CMV) test results for 5-point scale variables.

| n_complete | p_raw | p_poly |
| --- | --- | --- |
| 4518 | 0.225 | 0.250 |

### Multicollinearity test

**Table S24.** Correlation matrix of trust-related variables.

|  | IT | TR | PE | IC |
| --- | --- | --- | --- | --- |
| IT | 1.000 | 0.230 | 0.041 | 0.026 |
| TR | 0.230 | 1.000 | 0.286 | 0.264 |
| PE | 0.041 | 0.286 | 1.000 | 0.648 |
| IC | 0.026 | 0.264 | 0.648 | 1.000 |

**Table S25.** Variance inflation factors (VIF) for multicollinearity assessment.

| Equation | VIF (min) | VIF (max) |
| --- | --- | --- |
| PR equation | 1.051 | 1.889 |
| WS equation | 1.054 | 1.118 |

# **Section S5.** Figures

### Age interaction (DL × Age → PR)


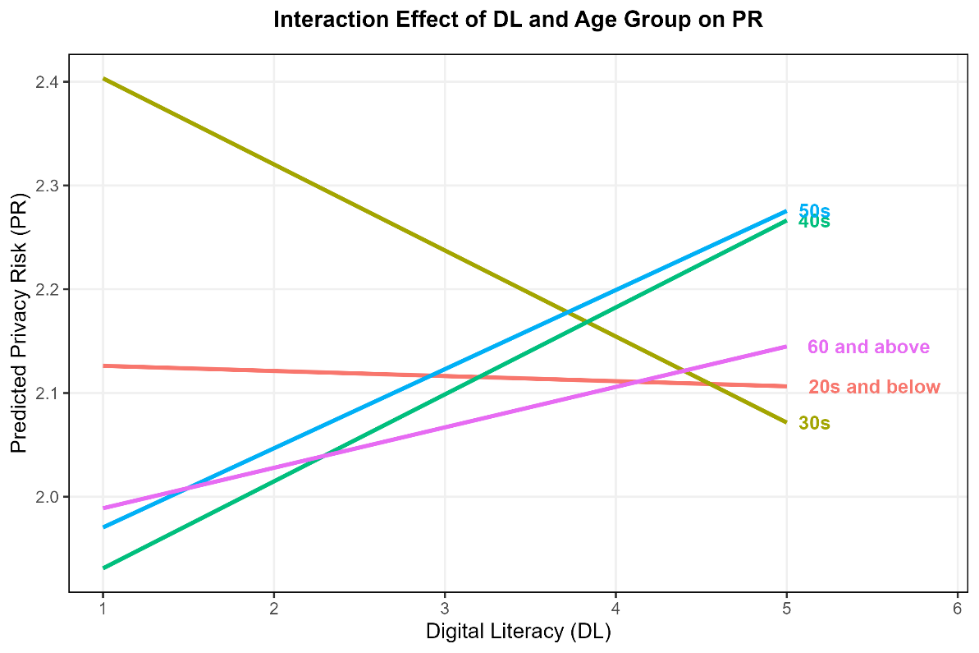


**Figure S1.** Interaction between digital literacy and age in predicting perceived risk (survey-weighted model). Lines represent predicted values of perceived risk across levels of digital literacy for each age group. Age was modeled as an ordinal variable.

### Independent Variables Distribution


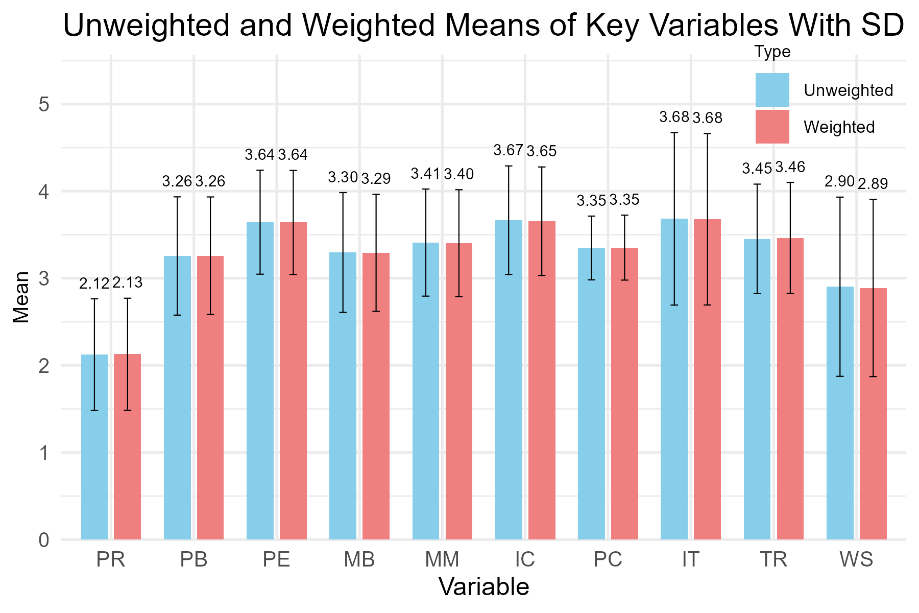


**Figure S2.** Unweighted and weighted means of key variables with standard deviations (SD). Bar chart illustrates the distribution of composite scores for main predictors used in the structural model.

Note: Error bars represent standard deviations (SD) for both unweighted and weighted estimates. Weighted statistics were calculated using survey weights. For constructs modeled as latent variables in the structural model (eg, PB and DL), item-averaged composite scores are presented to provide interpretable descriptive statistics on the original Likert scale. DL and its subdimensions were measured on 5-point Likert scales, whereas perceived risk (PR) was measured on a 4-point Likert scale. For PB, descriptive statistics are based on the mean of its observed indicators (q17_8, q17_13, q17_14).

### Study Model for H9 (Model 2)


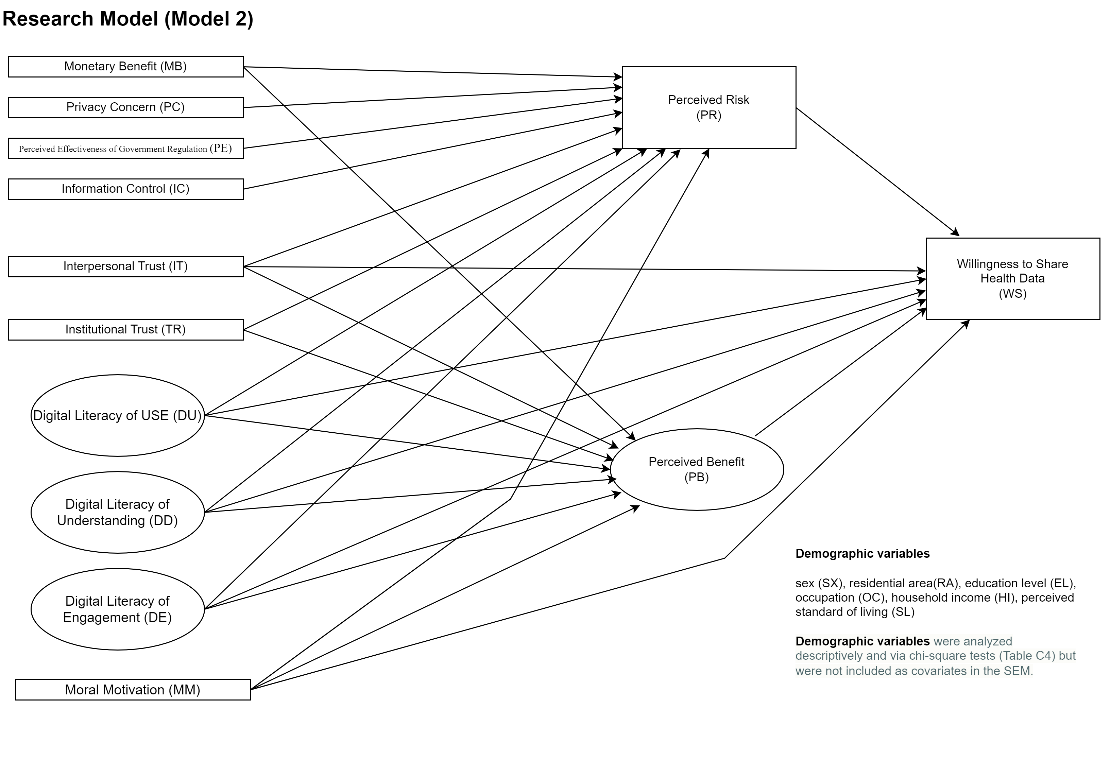


**Figure S3.** Structural model for Hypothesis 9 (Model 2) illustrating differential effects of digital literacy subdimensions.

### Result for Model 2 Weighted model


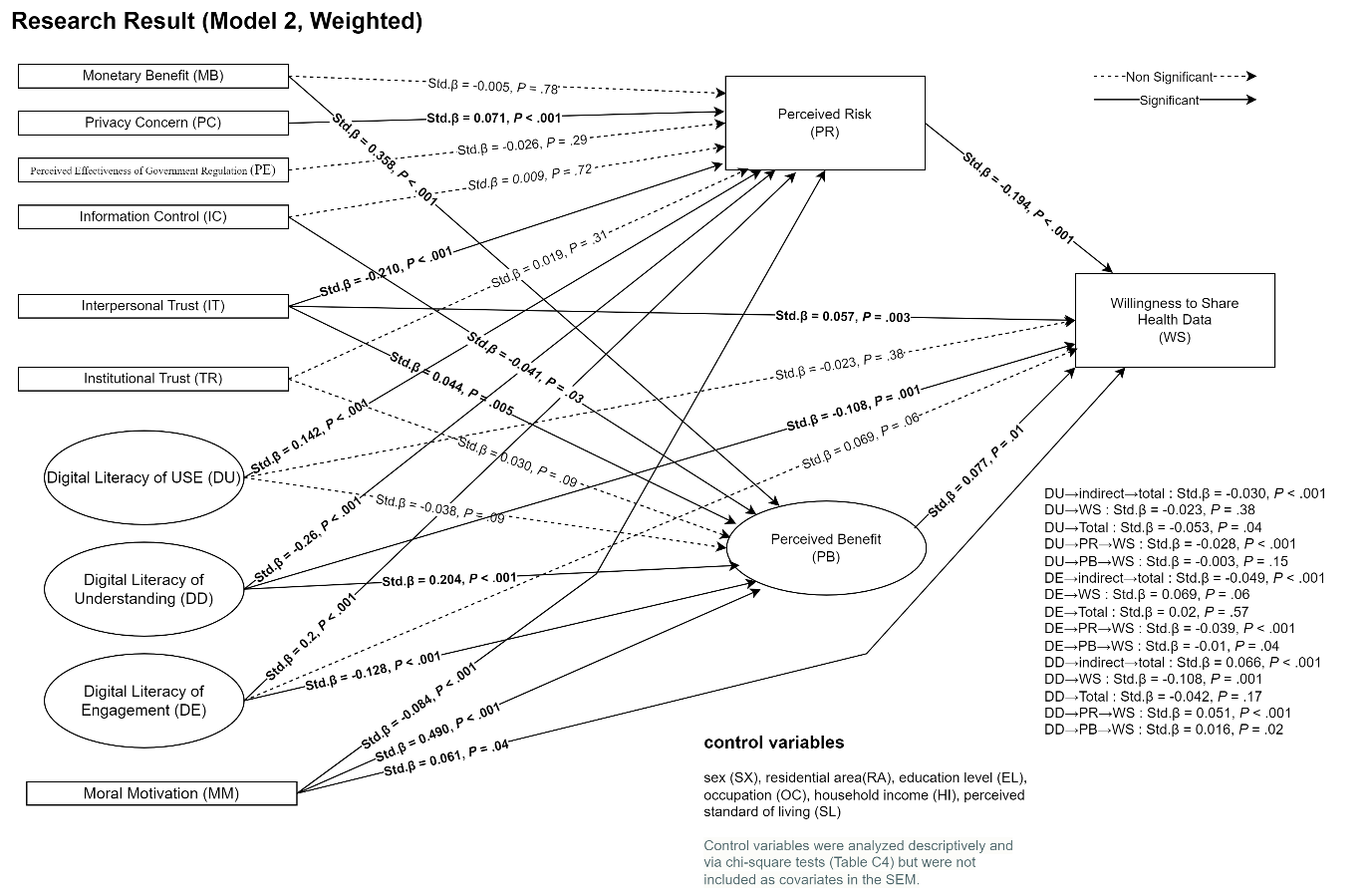


**Figure S4.** Standardized path coefficients for Model 2 (survey-weighted structural equation model). Solid lines indicate statistically significant paths (P < .05), and dashed lines indicate non-significant paths.
